# Curriculum Innovation: Teaching Neuroethics Through a Case-Based Undergraduate Medical Education Workshop: Implementation and Evaluation

**DOI:** 10.1212/NE9.0000000000200070

**Published:** 2023-05-15

**Authors:** Maya Overby Koretzky, Katemanee Burapachaisri, Bernadette Clark, Michael R. Halstead, Charlene E. Gamaldo, Rachel Marie E. Salas, Doris G. Leung, Carlos G. Romo

**Affiliations:** From the Department of the History of Medicine and Medical Scientist Training Program (M.O.K.), Johns Hopkins School of Medicine; Department of Neurology (M.O.K., K.B., B.C., C.E.G., R.M.E.S., D.G.L., C.G.R.), Johns Hopkins School of Medicine, Baltimore MD; Sentara Medical Group (M.R.H.), Norfolk, VA; and Kennedy Krieger Institute (D.G.L.), Baltimore, MD.

## Abstract

**Background and Problem Statement:**

Medical students on their clinical neurology clerkship often encounter ethically challenging situations, yet formal neuroethics training is limited. This study sought to evaluate a case-based small-group workshop that was implemented to introduce students to important neuroethics concepts and resources.

**Objectives:**

(1) To define decision-making capacity and describe how it is assessed in neurologic illness; (2) to define the legal category of brain death and its evolution over time; (3) to describe the legal process for surrogate decision making in the state of Maryland; (4) to identify barriers to goals-of-care conversations; and (5) to reflect on how personal beliefs of patients and physicians influence delivery of care and medical decision making.

**Methods and Curriculum Description:**

A 1.5-hour interactive curriculum for medical students on the neurology clerkship covering ethical considerations in brain death, surrogate decision making, and navigating conversations surrounding these topics (with reference to lesbian, gay, bisexual, transgender, queer/questioning, and others' health and health disparities) was designed and implemented over 2 years. Curriculum outcomes were measured by preworkshop and postworkshop self-assessment surveys. Learner reactions were measured by self-reported interest in ethics and perceived utility of the curriculum. Content knowledge was measured through multiple-choice questions on brain death and capacity assessment, which were scored for correctness by the study team, and self-reported confidence in ethical reasoning. Changes in these metrics were analyzed for paired precourse and postcourse responses to determine the effectiveness of the session.

**Results and Assessment:**

The study recruited 234 of 356 rotating students (65.7% response rate). Presurvey data revealed that 36% had encountered a challenging clinical scenario before the intervention. Preintervention and postintervention paired data were available for 184 (79%) respondents. Of these, 66% reported increased confidence in their knowledge of ethics, and 42% reported increased interest in ethics. Presession performance on content questions did not differ significantly based on prior clinical ethics experience. Performance on neuroethics content questions improved significantly after the session as demonstrated by the increase in the percentage of students providing correct answers to content questions between the presurvey and postsurvey (17% increase for capacity assessment, 19% increase for brain death, and 22% increase for surrogate decision making, *p* < 0.0001).

**Discussion and Lessons Learned:**

An interactive neuroethics workshop using a case-based discussion format integrates ethics and health disparity education into the clinical neurology curriculum and enhanced knowledge and confidence in medical ethics. This curriculum increases student interest in ethics, confidence in their ability to perform ethical reasoning tasks, and content knowledge of brain death and surrogate decision making.

## Introduction and Problem Statement

Education in medical ethics occurs at all levels of medical training in both formal and informal contexts. The Liaison Committee on Medical Education has designated medical ethics as a standard component of curricular content for undergraduate medical education.^1^ Beyond medical school, education in clinical ethics for neurology residents is recommended. The American Academy of Neurology (AAN) has developed an ethics curriculum for neurology residents,^2^ which has been used previously in a study of case-based ethics instruction at a major northeastern teaching hospital.^3^ The results of the study suggested that case-based ethics education was beneficial in increasing neurology residents' confidence and skill in approaching ethical issues. Unfortunately, such a resource does not yet exist for medical students in their neurology clinical clerkship.

The medical school core clerkship in neurology provides a unique opportunity for impactful neuroethics education. Research has shown that medical students frequently encounter ethically challenging situations through the hidden curriculum while on their clinical rotations,^4^ and that didactic ethics instruction in undergraduate medical education improves medical students' ability to navigate ethical challenges.^5,6^ Over the course of their neurology core clerkship, medical students have been shown to be regularly exposed to difficult conversations around ethical issues at the end of life, including brain death, prognostication, and assessment of capacity for decision making in a variety of clinical scenarios.^7^ Although these clinical scenarios are of wider interest to physicians in all specialties and to the general public, brain death and capacity assessment are often misunderstood by physicians and the lay public.^8-10^ In addition, over the past several years, there has been a documented need for greater diversity in neurologic education,^11,12^ including discussion of issues specific to lesbian, gay, bisexual, transgender, queer/questioning, and others (LGBTQ+) neurologic care^13^ and discussion of how the history of medical racism in America may affect how families of different cultural backgrounds respond to and understand a brain death diagnosis.^14^ As such, undergraduate medical education in neurology should include these topics; however, data are scarce regarding the best way to integrate ethical issues and difficult conversations into a clinical neurology curriculum.

The overall aim of this study was to develop and evaluate a case-based small-group ethics workshop for medical students that covered brain death and capacity assessment as part of the clinical clerkship in neurology at the Johns Hopkins School of Medicine. It was hypothesized that, after this activity, students' confidence in and content knowledge of medical ethics would increase. It was also hypothesized that this intervention would be useful even for students who already had practical experience with ethics scenarios in clinical settings.

## Objectives

An interactive case-based curriculum was designed to provide students with an opportunity to learn more about ethical issues in neurologic care, to consider how their own values may influence the conversations they have with patients, and to identify strategies for conducting difficult conversations—on quality of life, prognostication, and end-of-life wishes—with patients and families. The objectives for the curriculum were shared with learners as follows:

1. To define decision-making capacity and describe concrete principles by which it can be assessed in the context of neurologic illness.
2. To define the legal category of brain death and give a brief overview of how the concept of brain death has evolved over time in clinical practice.
3. To describe the legal process for surrogate decision making in the state of Maryland, with and without a preexisting advance directive.
4. To identify barriers to early and thorough goals-of-care conversations in the context of neurologic illness and discuss strategies to navigate such discussions with patients and families.
5. To reflect on how personal beliefs of both patients and physicians related to quality of life may influence delivery of care and medical decision making at various points in the course of a progressive neurologic disease or at the end of life.

## Methods and Curriculum Description

### Standard Protocol Approvals, Registrations, and Patient Consents

The protocol was reviewed and approved by the Johns Hopkins Medicine Institutional Review Board. Informed consent was obtained from all participants before enrollment in the study.

### Curriculum Description

All 356 students in the neurology clerkship at Johns Hopkins School of Medicine between August 2019 and August 2021 attended a neuroethics workshop, structured as a short lecture followed by 2 clinical cases. A case-based learning approach was selected because it is a well-established modality for teaching medical ethics.^15^ Cases were designed around a flipped classroom model, in which students learn the lesson content through prework and recorded lectures watched before the session and then use the in-class time to apply their knowledge through discussion with their peers in a synchronous classroom environment. This model has been shown to be effective in other medical school clerkship settings.^16,17^ The first case covered capacity assessment and advanced care planning in a person with impaired cognition and the order of surrogacy in the state of Maryland. The second case covered the historical, clinical, legal, and ethical aspects of brain death, highlighting brain death standards in the state of Maryland and at our own institution, while also emphasizing that legal criteria for a brain death diagnosis vary considerably state by state (the full text of the cases, with accompanying notes for facilitators, is available in eAppendix 1, links.lww.com/NE9/A23). The cases also asked students to reflect on the ways patient identity, including racial identity and sexual orientation, might affect discussion with families around these neuroethics topics.

Before March 2020, workshops were conducted in person over a period of 1.5 hours during the neurology clerkship block. Sessions were facilitated by a diverse group of educators, including peer teachers (fourth-year medical students), graduate students in bioethics and history of medicine, and neurology residents. All facilitators received a standardized training and curriculum packet, which included cases, background information, and suggested discussion prompts. In the spring of 2020, the Johns Hopkins School of Medicine switched to an online didactic curriculum in accordance with safety precautions related to the coronavirus disease 2019 pandemic. As online modalities have been shown to be effective for small-group education,^18^ the neuroethics workshop was converted to a hybrid synchronous/asynchronous activity, with approximately half an hour of e-lecture and prereading material before an hour-long case-based workshop conducted in small groups on Zoom (10–15 students per group). Because of this unexpected change in the delivery method and timing of the workshop, online and in-person data sets were analyzed for significant differences between groups before merging the data sets. This analysis is presented in the discussion.

The prework for the session included a review article on capacity assessment and surrogate decision making,^19^ a historical New York Times article from 1968 discussing the rise in organ transplantation in the context of the new medical category of brain death,^20^ and an article in the popular press covering a high-profile brain death case, including racial dimensions of the case.^21^ The e-lectures featured an overview of decision-making capacity considerations^22^; discussion of the order of surrogacy and legal considerations in Maryland, with a particular focus on patients with LGBTQ+ identities and diverse family structures^23^; a short history of brain death, organ transplantation, and medical racism^24^; a clinical review of the AAN brain death determination checklist; and discussion of differing legal aspects of a brain death diagnosis across the United States.^25^

Facilitators for the Zoom small-group discussions included neurology residents, faculty, and fellows; advanced medical students serving as teaching assistants for the neurology clerkship; and graduate students from the Berman Institute of Ethics and the Department of the History of Medicine. All facilitators were provided with standardized instructions for leading the workshop, including the full text of the cases broken into sections for discussion, instructions for how much time to spend on each section of a case, and suggested discussion prompts.

### Study Design

Although all clerkship students attended the workshop, only students who consented to participate in the study completed a presurvey and a postsurvey immediately before and after the ethics workshop to assess their level of interest in and knowledge of neuroethics issues (the full text of these surveys is included as eAppendix 2, links.lww.com/NE9/A23). The preworkshop survey asked students to report their previous exposure to clinical ethics and the number of clerkships completed before the ethics workshop. These questions were used to determine whether prior exposure to ethical issues in the hospital or classroom would affect students' responses and to gauge the timing of highest exposure to real-life ethical issues in a clinical context while on training. The presurvey and the postsurvey asked students to rate their level of agreement with the statement “I am confident in my knowledge of medical ethics” on a Likert scale ranging from 0 to 10 (with 0 being complete disagreement and 10 being complete agreement). Students' interest in medical ethics and perceived need for formal education in ethics were similarly scored on a scale of 0–10. The postsurvey also asked students to rate how strongly they agreed or disagreed that each section of the activity was useful for their education (capacity assessment, surrogate decision making, and brain death) on a 0–10 Likert scale (with 0 being complete disagreement and 10 being complete agreement). Both the presurvey and postsurvey included the same 3 questions designed to assess content knowledge regarding brain death, capacity assessment, and legal order of surrogacy in Maryland. Questions were multiselect multiple choice, meaning that a correct answer required students to select all the correct answer choices and none of the incorrect answer choices from a bank of options. The presurvey and the postsurvey completed by the same student were matched for metrics that required paired analysis (see statistical methods below). Students were also provided with space to leave qualitative comments about the session.

### Statistical Methods

An exploratory analysis of each variable was performed using tables and histograms. For variables that were scored on a scale of 1–10, Shapiro-Wilk testing was performed to determine whether variables met the assumption of normality. Pearson χ^2^ tests were used to compare ordinal and categorical variables. Paired *t* tests and Wilcoxon signed-rank tests were used to analyze the differences in preworkshop and postworkshop scores of confidence and interest in medical ethics, as well as the need for formal medical ethics training. The answers to content questions about brain death and capacity assessment were coded as incorrect, partially correct, or completely correct. For the purposes of estimating effect size of the intervention, 2 groups were compared: fully correct vs partially correct and completely incorrect. The McNemar test was used to compare preworkshop and postworkshop performance on content questions. The statistical significance threshold was set at <0.05 with a Bonferroni correction for multiple comparisons.

### Data Availability

At reasonable request from any qualified investigator, the authors will comply with providing deidentified data as long as approval by the Johns Hopkins Institutional Review Board is obtained.

## Results and Assessment Data

### Participants and Descriptive Data

Of the 356 students rotating in the neurology core clerkship at Johns Hopkins School of Medicine from August 2019 to August 2021, 234 agreed to participate and completed the presurvey for a response rate of 65.7%. The distribution of participants was 102 second-year, 80 third-year, and 52 fourth-year students. The investigators collected 234 preworkshop surveys and 209 postworkshop surveys. The follow-up survey was not completed by 50 students (21%). The baseline demographics of the survey participants are summarized in Table 1. Students who did not respond to the presurvey or failed to complete the postsurvey did not report any specific reasons for their lack of participation.

**Table 1:** Baseline Characteristics of Presurvey Respondents

	No. of second-year students	No. of third-year students	No. of fourth-year students
Format			
In person	0	23	13
Online	102	57	39
Encountered an ethically challenging situation			
No	95	47	8
Yes	7	33	44
Exposure to end-of-life/goals-of-care discussions			
No exposure	99	49	5
Witnessed	2	25	29
Participated	0	5	4
Witnessed and participated	1	1	14
Exposure to capacity assessment for clinical decision making			
No exposure	102	58	15
Witnessed	1	18	26
Participated	0	2	8
Witnessed and participated	0	2	3

### Prior Clinical Ethics Experience

All students were assumed to have experienced prior formal education because the preclinical curriculum at Johns Hopkins includes several ethics modules. On the presurvey, students were asked questions to determine practical clinical ethics experience. Thirty-six percent of respondents reported having encountered an ethically challenging scenario during their clinical training, and 35% had witnessed or participated in a conversation regarding end-of-life or a goals-of-care discussion. Students were also asked whether they had witnessed or participated in a capacity assessment for clinical decision making, and 26% reported prior experience. They were also asked to respond affirmatively or negatively to the statement: “I have encountered situations on my clinical clerkships that I have found ethically challenging.” A minority of second years responding yes to 1 or more of these 3 questions and the majority of fourth-year students responding affirmatively to all 3 questions.

Content knowledge questions on the presurvey were analyzed to determine whether prior clinical experience with specific ethics situations was associated with better performance on related content questions. A χ^2^ analysis showed that students who had reported prior experience with end-of-life conversations (witnessed or participated) did not score higher on the question regarding brain death compared with students who did not have these experiences. Among students who reported prior experience, 33% had an incorrect answer on the brain death question, 52% had a partially correct answer, and 15% had a fully correct answer; among students who did not report prior experience, 33% had an incorrect answer, 40% had a partially correct answer, and 27% had a fully correct answer (*p* = 0.08). Students who reported witnessing or participating in a capacity assessment did not score higher on the capacity assessment question than those who did not report these experiences. Among students who reported prior experience in capacity assessment, 20% had an incorrect answer, 45% had a partially correct answer, and 35% had a fully correct answer on the capacity question; among students without prior experience, 13% had an incorrect answer, 39% had a partially correct answer, and 48% had a fully correct response (*p* = 0.17). Students who reported having encountered an ethically challenging scenario on the wards did not score higher on any content question than students who did not report encountering an ethically challenging scenario on the wards. Interest in, confidence in, and belief in the importance of formal ethics education did not differ significantly based on prior clinical ethics experience.

### Content Knowledge on the Presurvey and Postsurvey

Analysis of the 184 students who completed both the presurvey and postsurvey indicated improved performance on the content knowledge questions after the workshop. On the capacity question, 48% of students answered correctly on the presurvey, whereas 65% answered the question correctly on the postsurvey (difference = 0.17; *p* < 0.0001; 95% CI 0.09–0.26). For the content question on brain death, 27% of students answered correctly on the presurvey, whereas 46% answered correctly on the postsurvey (difference = 0.19; *p* < 0.0001; 95% CI 0.12–0.27). For the question on surrogate decision making, 33% of respondents answered the question correctly on the presurvey, whereas 55% answered correctly on the postsurvey (difference = 0.22; *p* < 0.0001; 95% CI 0.14–0.30).

### Subjective Measures on the Presurvey and Postsurvey

Before the workshop, respondents' mean score of their confidence in their knowledge of medical ethics was 5.43 (SD 1.70, range 1–10); this level increased to 6.63 (SD 1.42, range 2–10) after the intervention (difference 1.20; *p* < 0.0001; 95% CI 0.91–1.50). In the same fashion, their degree of interest in medical ethics was measured. The prestudy assessment showed that the mean level of interest was 6.91 (SD 2.06, range 1–10) at baseline and increased to 7.46 (SD 1.95, range 2–10) in the postintervention questionnaires (difference = 0.55; *p* = 0.0043; 95% CI 0.17–0.93). Finally, students were asked to rate their perceived level of need for formal education in medical ethics as part of the medical school curriculum (0 was considered to be “no need for formal training,” and 10 was “all physicians need to have formal training”). Of the respondents, 73% gave this question a score of 8 or higher on the presurvey, whereas 82% gave a score of 8 or higher on the postsurvey (Table 2).

**Table 2:** Distribution of Likert Scale Ratings by Question on the Presurvey and Postsurvey^a^

Scores (0 = disagree completely; 10 = agree completely)	Presurvey, n (%)			Postsurvey, n (%)			*p* Value
	Mostly disagree (0–3)	Moderately agree (4–7)	Mostly agree (8–10)	Mostly disagree (0–3)	Moderately agree (4–7)	Mostly agree (8–10)	
I am confident in my knowledge of medical ethics	27 (15)	142 (78)	14 (8)	3 (2)	130 (71)	51 (28)	<0.001
I am interested in medical ethics	9 (5)	98 (54)	76 (42)	8 (4)	81 (44)	95 (52)	<0.001
I think all physicians should have formal medical ethics education during medical school	2 (1)	45 (25)	136 (74)	3 (2)	30 (16)	151 (82)	<0.001
I learned something in this activity that I feel I will use during clinical rotations/my future career as a physician				4 (2)	54 (26)	151 (72)	
The capacity assessment section was helpful to me				3 (2)	62 (30)	139 (68)	
The surrogate decision-making section was helpful to me				3 (2)	63 (31)	138 (68)	
The brain death section was helpful to me				2 (1)	49 (24)	153 (75)	

a *p* Values were calculated using paired *t* tests for the questions about confidence in medical knowledge interest in medical ethics and Wilcoxon signed-rank tests for the question about the need for formal medical ethics education.

The mean rating of the helpfulness of the capacity assessment section was 8.27 (SD 1.79). The mean rating for the surrogate decision-making section was 8.22 (SD 1.79), and the mean rating for the brain death section was 8.46 (SD 1.68). In response to the statement, “I learned something in this activity that I feel I will use during clinical rotations/my future career as a physician,” 72% of respondents gave this question a score of 8 or higher. In response to the statement, “the capacity assessment section was helpful to me,” 68.1% of respondents gave this question a score of 8 or higher. 67.6% of respondents reported a score of 8 or higher for the statement, “the surrogate decision-making section was helpful to me,” and 75% of respondents reported a score of 8 or higher for the statement, “the brain death section was helpful to me” (Table 2).

### Qualitative Perceptions of Activity

Written feedback for the activity was optional, but comments that were provided by students were overall positive, with critical feedback in the minority. Students who provided written feedback praised the small-group format and commented on the importance of discussing the intersection of racial, cultural, and LGBTQ+ identity with ethical issues in neurology. Critical comments were mostly focused on the instructional design rather than the content of the workshop and indicated that the quality of the session was greatly dependent on the quality of the facilitator and on the group dynamics. In terms of suggestions for the improvement of the workshop, several students expressed more interest in brain death over capacity assessment, suggested that the session be extended to include more time for students to work through brain death and capacity assessment checklists in conjunction with clinical cases, and had mixed feedback as to whether the session would have been improved by being in person rather than online. Representative qualitative comments are included in eAppendix 3 (links.lww.com/NE9/A23).

## Discussion and Lessons Learned

The objective of this study was to evaluate the utility of a case-based small-group workshop in teaching medical students about 2 important and widely applicable issues in neuroethics: decision-making capacity and brain death. In addition, the workshop aimed to increase students' interest and confidence in approaching neuroethics topics and to expose students to a diversity of patient identity considerations through clinical cases. The case-based workshop was a success across all the metrics we investigated: correct answers on content questions increased significantly after the intervention, as did students' subjective ratings of their own interest in medical ethics and confidence in their ability to perform ethical reasoning tasks. Students commented verbally during the workshop and in optional written feedback to praise the discussion-driven, case-based format of the activity and also expressed appreciation for how the activity addressed LGBTQ+ identity considerations and medical racism as important topics for health care providers. Including diverse patient identities in the clinical cases provided a model for integrating diversity into clinical neurologic education, as advocated by other researchers^13^ and by the AAN.^12^

Limitations of the study were introduced by the switch to virtual education in March 2020, necessitating the training and use of multiple facilitators for concurrent virtual sessions. We limited discrepancies between groups led by different facilitators by providing consistent training for all facilitators and by using the same e-lectures and prereading for each group. Although standardization of the facilitator role was the goal for this study, a strength of this curriculum was the recruitment of facilitators from a wide variety of backgrounds (medical humanities, medical ethics, clinical neurology, medical student peers). Facilitators were encouraged to share their own unique experiences and knowledge base rather than trying to follow a rigidly standardized curriculum.

Differences in responses between the online and in-person groups were explored for each of the variables collected in the presurvey and postsurvey. No statistically significant difference in the content and subjective measures was detected after adjusting for multiple comparisons. One possible reason that no differences were detected between these groups is the discrepancy in group size. Because only 36 students of 234 completed the session in person, this group is relatively underpowered to detect modest differences. There were significant differences between the online and in-person groups in questions dealing with prior experience (i.e., having witnessed or participated in challenging ethical situations, end-of-life care, or capacity assessments). However, this difference is almost certainly being driven by the fact that 2nd-year medical students made up 52% of the online group and none of the in-person group. This disparity was due to pandemic-related changes in the curriculum, which caused the session to be moved into the 2021 “Transition to the Wards” course, which is composed of students who had not started clinical rotations and had fewer opportunities to experience these clinical scenarios.

Additional limitations include the demographics of the study and response rate, use of an agreement scale rather than a confidence scale, and lack of follow-up to study the impact of the curriculum on clinical practice. The overrepresentation of second-year and third-year students over fourth-year students in the data limits the comparisons between students grouped by year. The response rate of the study (65.7%) is typical for voluntary educational survey research. The most likely explanation for the students who did not agree to participate is that they did not want to spend time on the survey. Some students also reported difficulty finding or accessing the presurvey, despite best efforts at providing clear instructions. The use of an agreement scale for assessing subjective beliefs about learner confidence may also have affected the data. Learner responses on agreement scales can be influenced by rater agreement, whereas a confidence scale could more accurately and optimally demonstrate a change in confidence. Although this limitation may have affected students' absolute ratings of the activity, the same scale was used for both the presurvey and postsurvey questions, ensuring that any observed change in learner beliefs was due to the activity rather than the survey instrument. Given that the postsurvey was administered directly after the workshop, it is not clear from this study what the educational effects of the workshop might be at more distant points in medical education. The study also does not assess translation of the content knowledge covered in the workshop to the behavior of doctors in training at the bedside and does not assess clinician behavior or patient outcomes.

Preworkshop survey scores on content questions were low, suggesting that the workshop provided information that was not included in the medical curriculum or covered informally. Although much research has been devoted to ethical challenges during residency training,^26^ few studies have considered how these experiences may shape medical training during undergraduate medical education. Despite the low scores on presurvey content questions, many students in their third or fourth year of medical school had previously encountered the topics covered in the workshop. The high percentage of fourth-year students who had encountered ethically challenging situations suggests that these experiences may arise earlier in medical training than previously appreciated. Our research also shows that hands-on experiences with ethically challenging situations in clinical training are not a substitute for formal medical ethics training. Students who reported practical clinical experience with ethics did not score better than their peers without practical experience on the content questions in the presurvey, suggesting that formal education can supplement and contextualize clinical exposure to ethical issues in medical education.

Although there was a statistically significant improvement in students' responses to content questions after the intervention, their overall performance—especially on the question regarding brain death—remained low after the workshop (46% of students answered the brain death question completely correctly on the postsurvey, whereas 65% of students answered the capacity assessment question completely correctly on the postsurvey). This performance may be low because the strictest possible standard for correctness was chosen in the analysis, and the study design did not give students any partial credit for selecting some but not all correct answer choices. Because there were so many different partially correct answers, analyzing responses based on exact answers would have resulted in splitting the sample into many groups that each contained relatively small numbers of respondents. For this reason, the measure of improvement chosen for this study most likely underestimates actual improvement.

In terms of the discrepancy between students' knowledge of capacity assessment and their knowledge of brain death, several qualitative comments on the activity mentioned that the brain death material was new for many students, whereas the capacity assessment and the order of surrogacy material overlapped with the psychiatry clerkship curriculum at our institution. This overlap is likely responsible for the higher overall performance on the capacity assessment questions (both before and after the workshop) and suggests that a good strategy to improve students' knowledge of brain death would be to provide several educational interventions covering the topic over the course of undergraduate medical education.

In contrast to their scores on content questions before the workshop, students' initial ratings of their interest in medical ethics and their belief in the importance of formal ethics education were quite high before the activity. This high rating of ethics content, even before the workshop, suggests that most medical students were eager to see more formal ethics education in the curriculum. This suggestion is supported by optional written comments after the activity in which students expressed their appreciation for its inclusion in the neurology curriculum.

Of interest, third-year medical students scored the lowest of all groups (second, third, and fourth years) in terms of their confidence in approaching ethics topics. Despite their low confidence, third-year students also reported extensive practical experience with ethics topics in the hospital setting. These data suggest that when students first encounter complicated ethics issues on the wards in practice (rather than in the classroom setting during their second year), they may lose confidence in their ethical reasoning abilities. Thus, ethics education would be the most beneficial early in their clinical clerkship experience.

This research has demonstrated that there is pedagogic utility in formal, case-based ethics instruction for students in the clinical years of medical school. In particular, it is important for clinical neurology instruction to cover topics such as brain death and capacity assessment, which are specialized issues in neurology and also widely applicable across medical fields and frequently discussed by the lay public. In the postsurvey, students rated the brain death case as particularly helpful. Survey research has demonstrated that the lay public by and large does not understand brain death,^9^ and that practicing physicians also often hold misconceptions about brain death,^10^ and even neurologists vary significantly in their approach to conducting and interpreting a brain death examination.^27^ For this reason, it is important to include dedicated brain death education in undergraduate medical education. Including a diversity of patient identities in the clinical cases used in this workshop also provided a model for how to integrate diversity into clinical neurologic education, as advocated by other researchers^13^ and by the AAN.^12^

In implementing this case-based workshop, several important lessons were learned. Although nearly all medical students are exposed to ethical issues on the wards over 4 years of training, supplementing this practical experience with a targeted ethics curriculum enables effective learning to occur. Recruiting facilitators from a variety of backgrounds exposes learners to new perspectives, whereas a standardized curriculum ensures that the same basic information is delivered across workshop sessions. Although the presurvey and postsurvey used in this study were effective for evaluating the overall success of the neuroethics curriculum, future researchers working with a similar study design may consider using a survey instrument with confidence rather than agreement scales for evaluating subjective metrics. Researchers may also prefer binary true-false or multiple-choice questions (rather than multiselect multiple-choice questions) to evaluate changes in content knowledge of learners in more granular detail. Overall, we hope that this study will serve as a model for clinical educators and clerkship directors looking to develop neurology-specific ethics curricula for medical students rotating on the wards. Our research shows that neuroethics topics are interesting to medical students, easily integrated into clinical clerkship curricula, and effectively taught through case-based instruction.

## Appendix Authors

**Table:**

Name	Location	Contribution
Maya Overby Koretzky, BA	Department of the History of Medicine and Medical Scientist Training Program, Johns Hopkins School of Medicine, Baltimore, MD	Drafting/revision of the manuscript for content, including medical writing for content; major role in the acquisition of data; study concept or design; and analysis or interpretation of data
Katemanee Burapachaisri, BS	Department of Neurology, Johns Hopkins School of Medicine, Baltimore, MD	Drafting/revision of the manuscript for content, including medical writing for content; and analysis or interpretation of data
Bernadette Clark	Department of Neurology, Johns Hopkins School of Medicine, Baltimore, MD	Major role in the acquisition of data
Michael R. Halstead, MD, MPH	Sentara Medical Group, Norfolk, VA	Study concept or design
Charlene E. Gamaldo, MD	Department of Neurology, Johns Hopkins School of Medicine, Baltimore, MD	Drafting/revision of the manuscript for content, including medical writing for content
Rachel Marie E. Salas, MD, MEd	Department of Neurology, Johns Hopkins School of Medicine, Baltimore, MD	Drafting/revision of the manuscript for content, including medical writing for content
Doris G. Leung, MD, PhD	Department of Neurology, Johns Hopkins School of Medicine; Kennedy Krieger Institute, Baltimore, MD	Drafting/revision of the manuscript for content, including medical writing for content; analysis or interpretation of data; and additional contributions: statistical analysis
Carlos G. Romo, MD	Department of Neurology, Johns Hopkins School of Medicine, Baltimore, MD	Drafting/revision of the manuscript for content, including medical writing for content; major role in the acquisition of data; study concept or design; and analysis or interpretation of data

## Glossary

AAN: American Academy of Neurology
LGBTQ+: lesbian, gay, bisexual, transgender, queer/questioning, and others
